# Complete Sequence and Annotation of the Mycoplasma phocirhinis Strain 852^T^ Genome

**DOI:** 10.1128/MRA.00131-19

**Published:** 2019-03-28

**Authors:** Salvatore Frasca, Jessy Castellanos Gell, Gerald F. Kutish, Dina L. Michaels, Daniel R. Brown

**Affiliations:** a Department of Comparative, Diagnostic, and Population Medicine, College of Veterinary Medicine, University of Florida, Gainesville, Florida, USA; b Department of Pathobiology and Veterinary Science and Center of Excellence for Vaccine Research, University of Connecticut, Storrs, Connecticut, USA; c Department of Infectious Diseases and Immunology, College of Veterinary Medicine, University of Florida, Gainesville, Florida, USA; University of Rochester School of Medicine and Dentistry

## Abstract

The genome of Mycoplasma phocirhinis strain 852^T^ was examined for determinants of tropism or virulence. It encodes multiple orthologs of an immunosuppressor that may predispose susceptibility to infection or influence outcomes of intercurrent diseases in marine mammals.

## ANNOUNCEMENT

Episodic seal die-offs documented along northern coasts of the Atlantic Ocean since 1979 have been attributed to morbillivirus or influenzavirus outbreaks involving pneumonia with systemic disease (1, 2). Mycoplasma phocirhinis was first isolated from the respiratory tracts and hearts from harbor seals (Phoca vitulina L.) that died along the adjacent North and Baltic seacoasts during a mass mortality event in 1988 (3–5). In order to understand better the role of M. phocirhinis in marine mammal mortalities and the implications of such events for human and ocean health, we sequenced and annotated the genome of type strain 852 from pus from a seal lung using the same approach we employed to examine the genome of Mycoplasma phocidae strain 105^T^ (6). Briefly, the DNA was extracted directly from a lyophilized specimen of M. phocirhinis (ATCC catalog no. 49639) using an Easy-DNA genomic DNA purification kit (Thermo Fisher Scientific catalog no. K180001). Libraries were constructed using the NEBNext UltraTMII DNA library prep kit (New England Biolabs catalog no. E7645S) and dual-index NEBNext multiplex oligos (New England Biolabs catalog no. E7600S) for Illumina MiSeq 2 × 150-bp paired-end sequencing. Trimming, adapter removal, and quality filtering with NxTrim v0.4.3 (7) yielded approximately 2.4 × 10^7^ reads with a PHRED score of >Q30. The final assembly, with a coverage depth of 250×, was achieved by using a combination of PEAR v0.9.11, Ray v2.3.1, Edena v3.131028, and Circlator v1.5.5 software (8–11) and then annotated starting at the *dnaA* gene via the RASTtk and NCBI Prokaryotic Genome Annotation pipelines (12, 13), all with default settings.

The closed circular genome is 865,472 bp in length, with G+C content of 27.5 mol%, close to the 26.5 mol% estimated by using isopycnic centrifugation (3). It is enlarged by 16 IS*3* and IS*1634* family insertion elements, in contrast to the single IS*3* element present in the 8.14-kbp genome of M. phocidae (6). Only about half of the predicted open reading frames (ORFs) have assigned functions, and only one-third could be grouped in subsystems, including structural RNAs; features of DNA, RNA, protein, carbohydrate, fatty acid, or phospholipid metabolism; transport or binding factors; and cell division factors. The presence of genes encoding cytosine 5-methyltransferase (CpG DNA methylase) and types I, II, and III restriction-modification system DNA methylases is evidence that the genome may undergo epigenetic modifications.

M. phocirhinis encodes three proteins (EG856_01490, EG856_01555, and EG856_02135) that have about 60% amino acid sequence similarity to mycoplasmal protein M, which binds the light chain of host IgG to block antigen-specific binding (14). These proteins may enable M. phocirhinis to evade host defenses or modulate intercurrent distemper or influenza in seals. Although adherence and cytotoxicity of M. phocirhinis to epithelial cells has been demonstrated in a tracheal explant model (15), no other determinants of tropism or virulence were discerned by genome analysis.

### Data availability.

The M. phocirhinis 852^T^ genome sequence and annotation have been deposited in GenBank as BioProject PRJNA505803. The raw data are available in NCBI's Sequence Read Archive as SRX5310557.
